# *t*-10, *c*-12 CLA Dietary Supplementation Inhibits Atherosclerotic Lesion Development Despite Adverse Cardiovascular and Hepatic Metabolic Marker Profiles

**DOI:** 10.1371/journal.pone.0052634

**Published:** 2012-12-20

**Authors:** Patricia L. Mitchell, Tobias K. Karakach, Deborah L. Currie, Roger S. McLeod

**Affiliations:** Department of Biochemistry and Molecular Biology, Dalhousie University, Halifax, Nova Scotia, Canada; Heart Center Munich, Germany

## Abstract

Animal and human studies have indicated that fatty acids such as the conjugated linoleic acids (CLA) found in milk could potentially alter the risk of developing metabolic disorders including diabetes and cardiovascular disease (CVD). Using susceptible rodent models (apoE^−/−^ and LDLr^−/−^ mice) we investigated the interrelationship between mouse strain, dietary conjugated linoleic acids and metabolic markers of CVD. Despite an adverse metabolic risk profile, atherosclerosis (measured directly by lesion area), was significantly reduced with *t-*10, *c*-12 CLA and mixed isomer CLA (Mix) supplementation in both apoE^−/−^ (p<0.05, n = 11) and LDLr^−/−^ mice (p<0.01, n = 10). Principal component analysis was utilized to delineate the influence of multiple plasma and tissue metabolites on the development of atherosclerosis. Group clustering by dietary supplementation was evident, with the *t*-10, *c*-12 CLA supplemented animals having distinct patterns, suggestive of hepatic insulin resistance, regardless of mouse strain. The effect of CLA supplementation on hepatic lipid and fatty acid composition was explored in the LDLr^−/−^ strain. Dietary supplementation with *t*-10, *c*-12 CLA significantly increased liver weight (p<0.05, n = 10), triglyceride (p<0.01, n = 10) and cholesterol ester content (p<0.01, n = 10). Furthermore, *t-*10, *c-*12 CLA also increased the ratio of 18∶1 to 18∶0 fatty acid in the liver suggesting an increase in the activity of stearoyl-CoA desaturase. Changes in plasma adiponectin and liver weight with *t-*10, *c-*12 CLA supplementation were evident within 3 weeks of initiation of the diet. These observations provide evidence that the individual CLA isomers have divergent mechanisms of action and that *t-*10, *c-*12 CLA rapidly changes plasma and liver markers of metabolic syndrome, despite evidence of reduction in atherosclerosis.

## Introduction

The relationship between diet and disease is of increasing importance as nutrient availability is increased and lifestyle changes put increased pressure on physiological mechanisms to maintain metabolic homeostasis [1], [2]. The incidences of obesity, type II diabetes and atherosclerosis are continually increasing and moreover, dietary choices, specifically dietary fat, may be a significant determinant in the development of cardiovascular and metabolic diseases [2]. Many fatty acids have been identified with biological properties that extend beyond simple nutrition and may have profound effects on normal physiology. Animal and human studies have indicated that the two major isomeric forms of conjugated linoleic acid (CLA) are biologically active, can have highly divergent physiological properties when administered individually, and do not necessarily have synergistic effects when consumed together [3]–[5]. Some reports support a role for CLA in decreasing body fat [6]–[8] and improving insulin action, predominantly in rodents (reviewed in [9], [10]). Other reports have indicated a lack of beneficial effects in rodents and humans [3], [11]–[14]. In addition, there is some evidence to support a role for CLA in the modulation of plasma lipid metabolism [15] and the development of atherosclerotic plaques (reviewed in [16], [17] ).

Cardiovascular diseases are a major cause of death in type 2 diabetic patients. In addition to coronary artery disease there is a direct adverse effect of diabetes on the heart, independent of other risk factors [18]. Insulin resistance occurs when insulin can no longer efficiently signal appropriate metabolic changes in target tissues, even though blood glucose levels are normal. Insulin resistance is one of the leading factors for the development of cardiovascular disease, stroke, non-alcoholic fatty liver disease, polycystic ovary syndrome, asthma and some cancers [19]. This lack of appropriate signalling results in abnormal cellular and plasma lipid metabolism that favours a proatherogenic state (reviewed in [20]). The liver is central to metabolic syndrome and understanding how hepatic physiology is altered at the cellular level may lead to a further understanding of the molecular mechanisms that signal the transition from simple stress (physiology) to distress (pathology). However, the mechanisms have not been clearly elucidated. Hepatic insulin resistance, as studied using the liver insulin receptor knockout mouse, has been shown to be sufficient to enhance very low density lipoprotein (VLDL) production and increase the risk of developing atherosclerotic lesions [21]. One of the initial changes in the insulin resistant state is an increase in circulating free fatty acids, possibly due to a decreased suppression of lipolysis in the adipocyte.

The development of murine models of atherosclerosis that mimic the human disease has allowed for the investigation into the mechanisms of CVD initiation and progression (reviewed in [22]). Previous work from our laboratory indicated that supplementation of high fat, high cholesterol (1.25%, w/w) diets with CLA isomers did not alter atherosclerosis in the apoE^−/−^ mouse [23]. However, in that work, the atherosclerotic lesions were very advanced, potentially masking the beneficial effects of CLA. In the current study we have explored the effects of individual and mixed isomer preparations of CLA on atherosclerosis and liver metabolism in two murine knockout models on high fat diets with a lower level of cholesterol (0.12%, w/w).

## Materials and Methods

### Ethics Statement

All procedures involving animals were approved by Dalhousie University Committee on Laboratory Animals in accordance with the guidelines of the Canadian Council on Animal Care.

### Animals and Diets

Male homozygous apoE^−/−^ or LDLr^−/−^ mice (8–10 weeks old), on a C57BL/6 background (Jackson Laboratories, Bar Harbour ME) were randomly assigned to five groups of 10–15 animals each and were fed, *ad libitum*, a semi-purified diet containing 16% (w/w) fat and 0.12% (w/w) cholesterol (Bio-Serv AIN-93G, Frenchtown, NJ) for 11 weeks. Control animals received the high-fat-cholesterol diet (HFC), while the diets of treatment groups were supplemented to 0.5% (w/w) with LA (Sigma-Aldrich, Oakville, ON), *c*-9, *t*-11 CLA, *t*-10, *c*-12 CLA or a 1∶1 mixture (Mix) of the two CLA isomers (Tonalin® FFA 80; 39% *c*-9, *t*-11 CLA/38% *t*-10, *c*-12 CLA). CLA was provided by Cognis Nutrition and Health and produced by Natural Lipids (Hovdebygda, Norway). Fatty acids were added to the base diet as an ethanol solution of the free acid [23], [24] and the ethanol was then removed by lyophilisation for 24–48 h. All diets were stored at -20°C until use, and were used within 2–3 weeks of preparation. Mice were housed 4–5 per cage and food consumption was measured every 3 days and recorded as average per animal.

Fatty acid composition of each diet was confirmed by gas chromatography analysis (Table S1) as described previously [23]. Briefly, total lipid extracts of the diet were prepared using a ratio of 18 parts solvent to one part diet. Phases were separated by centrifugation following the addition of six parts 0.9% NaCl. Lipids were dried from the chloroform phase under nitrogen and hydrolyzed using 1 N KOH in ethanol to release free fatty acids, which were then extracted using diethyl ether/hexane. Methylation of the free fatty acids was carried out according to Yurawecz [25].

### Quantification of Atherosclerosis

Mice were anaesthetized by inhalation of isoflurane. Once complete anaesthesia was achieved, exsanguination by ventricular puncture was performed. The right atrium was then nicked and the vasculature was perfused with 10 mL PBS by injection into the puncture site at the apex of the left ventricle. The heart and aorta, from the aortic arch to the iliac bifurcation, were excised, and fixed for quantification of atherosclerosis as previously described [23].

### Measurement of Blood Glucose, Lipids and Lipoproteins

Fasting blood was collected via ventricular puncture into syringes containing 2 µL of 0.5 M EDTA solution. A 5 h fast was used to minimize the metabolic stress that can result from prolonged fasting [26]. Blood glucose concentrations were measured immediately using an Accu-chek® Advantage Meter (Roche Diagnostics, Quebec City, QB). Plasma was isolated by centrifugation at 15,600 x g for 10 minutes at 4°C and then refrigerated prior to density gradient ultracentrifugation. For plasma lipid and protein analyses, aliquots of plasma were stored frozen at −80°C.

Lipoproteins were resolved as previously described [23]. In brief, plasma samples from five animals per treatment group were pooled to obtain 1000 µL total volume and sucrose was added to a final concentration of 12.5% (w/v). A discontinuous sucrose density gradient was constructed by underlayering 1 mL of phosphate buffered saline solution (PBS) with the sucrose-plasma sample, and 670 µL each of 25% and 47% sucrose solution (in PBS). Lipoproteins were separated by ultracentrifugation at 310,000 x *g* for 20 h at 12°C in a SW60Ti rotor. Twenty fractions of 200 µL each were then collected from the top of the centrifuge tube and cholesterol and triglycerides were measured in the fractions as described below. Sucrose was used as the gradient material as it does not interfere with subsequent lipid analyses and therefore does not require the fractions to be dialysed.

Cholesterol and triacylglycerol concentrations in plasma samples and gradient fractions were determined by enzymatic assays using reagents from Roche Diagnostics or Sigma-Aldrich, respectively, adapted for microtiter plate format. For cholesterol assays aliquots of 1.25 µL (plasma) and 2.5 µL (density fractions) were analysed. For triacylglycerol analysis, aliquots of 5 µL (plasma) and 25 µL (density fractions) were analysed. The lipoproteins were defined by density of the fractions; VLDL (1–4, ρ = 1.02–1.04 g/mL), LDL (5–10, ρ = 1.04–1.07 g/mL), HDL (11–20, ρ = 1.07–1.23 g/mL).

### Measurement of Plasma Adiponectin, Insulin and Non-esterified Fatty Acids (NEFA)

Plasma adiponectin and insulin were measured by enzyme-linked immunosorbent assay kits (EZMADP-60K and EZRMI-13K, respectively, Linco Research, St Charles, MO) according to manufacturer’s instructions. Plasma NEFA were measured using an enzymatic assay formatted for microtiter plates (ACS-ACOD MEHA, WAKO Chemicals USA, Richmond, VA).

### Measurement of Liver Lipids

Following exsanguination, the liver was excised, weighed, and immediately frozen in liquid nitrogen. The tissue was stored at -80°C until analysis. Total lipid was extracted from a portion of each liver according to Folch *et al.* [27], as described above for analysis of diet fatty acids. Phases were separated by centrifugation following the addition of 6 parts 0.9% NaCl. Total lipid in extract was determined by weight after drying the chloroform phase under nitrogen into tared glass tubes. Internal standard (tricaparin) was added to an aliquot of lipid extract and the lipid species were derivatized with bis(trimethylsilyl)triflouroacetamide (BSTFA) at room temperature for 1 h. Lipid classes in the sample were quantified using temperature-programmed gas-liquid chromatography on a Hewlett-Packard 6890 Capillary FID gas chromatograph fitted with a 30 m column (0.32 mm i.d.) coated with crosslinked 5% Ph Me silicone (0.25 µm film thickness; Hewlett-Packard HP-5; Palo Alto, CA) attached to a 0.53 i.d. retention gap [23].

### Statistical Analysis

Analysis of variance (ANOVA) was used to determine differences between the HFC group and supplemented groups for phenotypic data, plasma measurements and liver metabolites. Data are presented as mean ± S.E.M. and differences were considered significant at p<0.05 as determined by Games Howell *post hoc* analysis (SPSS for Windows, Version 17.0). This analysis was selected as powerful and accurate when sample sizes are unequal and the homogeneity of variance assumption has been violated.

Principal component analysis (PCA) is an approach for exploratory data analysis and visualization that allows patterns in data tables of complex measurements (consisting of observations quantitatively described by several auto-correlated variables) to be discerned. The theory and principles of PCA have been extensively covered in standard text books [28] while recent applications in biology have become common [29]–[31]. The goal of PCA is to mathematically project such complex multidimensional data onto the space of a new orthogonal coordinate system described by variables obtained as linear combinations of the original data. These variables are referred to as principal components (PCs) and are, as well as being linear combination of original variables, obtained by maximizing the data variance. Each new PC describes the part of the data variance not modeled by its predecessor and is added as a column or row to a matrix of PCs in order of decreasing significance (i.e., PC1>PC2>PC3 etc.). Frequently, projection of the observations onto the space defined by the first two or three PCs displays a pattern of (dis)similarity of the observations and variables as points in a dimension that is easier to integrate by the human eye.

For PCA, the measured data were pre-processed as follows. The matrix **X**_(m x n)_, *m* variables by *n* animals, were normalized to unit sum and sphered (*i.e*., set to a column mean of zero and unit variance) before analysis via singular value decomposition (SVD) in MATLAB 7.11.0 (MathWorks®, Natick, MA). PCA was then used to explore the relationship among the mouse strains based on the dependent variables.

## Results

### CLA – associated Body and Liver Weight Changes are Isomer Specific

The initial within strain body weights of animals did not differ between dietary treatment groups (Table 1). Food consumption during the study was also not different within strain. The *t*-10, *c*-12 CLA supplemented dietary groups gained less weight in both mouse strains (4.2±0.5 g in apoE^−/−^ and 4.7±0.5 g in LDLr^−/−^) than the control groups (p<0.001 and p<0.05, respectively; Table 1). Concomitant with the changes in body weight, liver weight increased with *t-*10, *c*-12 CLA and CLA Mix supplementation. This increase was significant in both absolute weight (not shown) and when expressed as a percentage of body weight (p<0.001 and p<0.05; apoE^−/−^ and LDLr^−/−^ respectively) (Table 1). Thus, inclusion of supplemental *t-*10, *c-*12 CLA in the diet reduced overall weight gain but induced significant hepatomegaly in both mouse strains.

**Table 1:** Phenotypic Characteristics of apoE^−/−^ and LDLr^−/−^ mice.

ApoE^−/−^	Diet Supplementation					
	HFC	LA		*c*-9, *t*-11 CLA	*t*-10,*c*-12 CLA	CLA Mix
Food Consumption(g/day/animal)	3.0±0.2	2.8±0.1		2.7±0.1	3.0±0.4	2.8±0.3
Initial BW (g)	23.9±0.5	24.6±0.4		23.1±0.6	23.8±0.8	22.1±0.5
WeightChange (g)	11.9±0.8	9.3±0.7		10.2±0.9	4.2±0.5***	9.0±0.7
Liver weight(% body wt)	5.6±0.3	4.9±0.4		5.4±0.3	12.1±0.6***	8.2±0.2***
**LDLr^−/−^**	**Diet Supplementation**					
	**HFC**		**LA**	***c*** **-9, ** ***t*** **-11 CLA**	***t*** **-10,** ***c*** **-12 CLA**	**CLA Mix**
Food Consumption(g/day/animal)	2.4±0.03		2.0±0.03	1.8±0.05	1.9±0.03	2.0±0.04
Initial BW (g)	18.6±0.6		18.2±0.3	17.2±0.6	16.9±0.5	17.6±0.6
WeightChange (g)	9.5±1.2		6.0±0.5	7.6±0.7	4.7±0.5*	5.7±0.6
Liver weight(% body wt)	3.7±0.2		3.5±0.1	3.8±0.1	8.7±0.5*	4.6±0.1*

Mice were fed a HFC diet either supplemented or not with 0.5% fatty acid for 11 wk. Food consumption was determined every 3 days. Liver weight was determined at sacrifice. Data are mean ± S.E.M. (n = 10 to 13). * p<0.05 vs. HFC, and *** p<0.001 vs. HFC.

### CLA – associated Changes in Plasma Metabolites are Isomer Specific

CLA isomers had complex effects on plasma lipids that were different in the two mouse models (Table 2). Plasma cholesterol was largely unaffected by dietary supplementation in the apoE^−/−^ mice but triglycerides were significantly lower in the *t-*10, *c-*12 CLA and mixed isomer CLA supplemented groups.

**Table 2:** Plasma Measurements for apoE^−/−^ and LDLr^−/−^ mice.

ApoE^−/−^	Diet Supplementation					
	HFC	LA		*c*-9, *t*-11 CLA	*t*-10,*c*-12 CLA	CLA Mix
Triglycerides (mM)	2.8±0.2	2.9±0.3		2.8±0.3	2.0±0.2*	1.3±0.2***
Cholesterol (mM)	32.9±1.0	34.5±1.8		39.0±2.6	36.3±3.7	28.9±2.0
Glucose (mM)	16.3±0.7	14.2±0.9		14.5±0.8	14.2±0.6	16.8±0.7
Insulin (ng/mL)	0.6±0.2	0.48±0.1		0.6±0.1	10.3±3.0*	1.61±0.8
Adiponectin (µg/mL)	11.2±0.5	11.3±0.8		9.3±0.6	0.8±0.1***	2.7±0.2***
NEFA (mEq/L)	0.48±0.03	0.46±0.04		0.42±0.02	0.5±0.06	0.37±0.03
**LDLr^−/−^**	**Diet Supplementation**					
	**HFC**		**LA**	***c*** **-9, ** ***t*** **-11 CLA**	***t*** **-10,** ***c*** **-12 CLA**	**CLA Mix**
Triglycerides (mM)	5.2±0.9		3.8±0.5	7.0±1.4	13.6±3.0	3.1±0.4
Cholesterol (mM)	21.5±3.0		16.8±1.7	31.3±2.6	43.0±4.6**	16.7±1.0
Glucose (mM)	10.5±0.6		9.5±0.5	10.6±0.7	13.6±0.9	12.6±0.9
Insulin (ng/mL)	0.6±0.1		0.5±0.1	0.5±0.1	7.1±3.2	0.8±0.1
Adiponectin (µg/mL)	13.8±2.4		13.1±1.1	13.4±0.7	1.6±0.2**	5.9±1.4
NEFA (mEq/L)	1.0±0.1		1.2±0.1	0.3±0.1^**^	0.8±0.1	0.5±0.1**

Mice were fed a HFC diet either supplemented or not with 0.5% fatty acid for 11 wk. Data are mean ± S.E.M. (n = 10 to 13). * p<0.05 vs. HFC, ** p<0.01 vs. HFC and *** p<0.001 vs. HFC.

In contrast, cholesterol was increased 2-fold by *t*-10, *c*-12 CLA supplementation in the LDLr^−/−^ mice (p<0.01, n = 10; Table 2). Changes with *c-*9, *t-*11 CLA supplementation in the LDLr^−/−^ mice were in the same direction as for the *t-*10, *c-*12 isomer, but did not reach statistical significance. Given that the individual isomers increased plasma cholesterol and TG, it was surprising that when supplemented as a mixed isomer, cholesterol and triglycerides in the LDLr^−/−^ mice were reduced compared to the control group (p<0.05).

**Figure 1:**
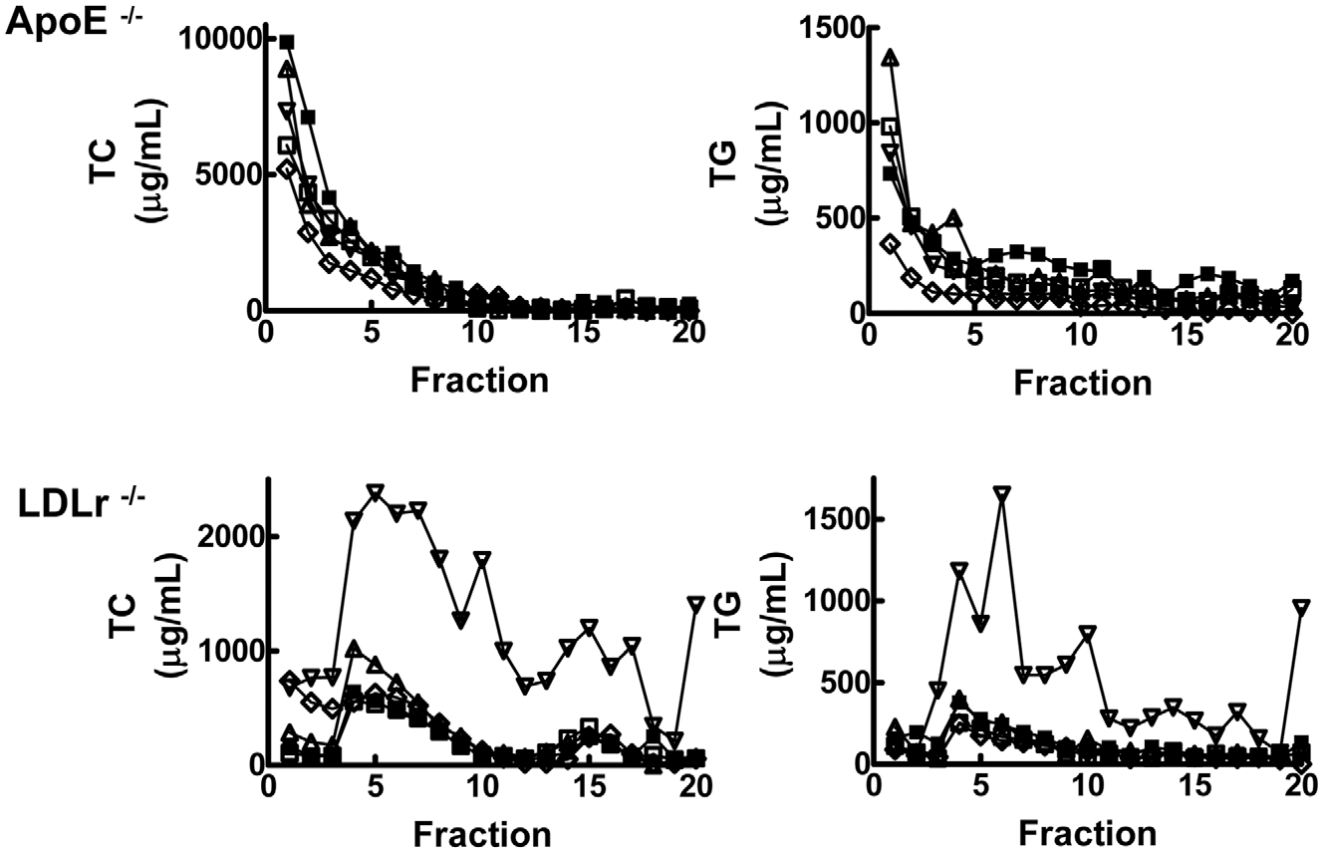
Plasma lipoprotein cholesterol and triglyceride profiles for apoE^−/−^ and LDLr^−/−^ mice. After 11 weeks on a HFC diet supplemented with 0.5% (w/w) of the indicated fatty acid, serum was obtained from apoE^−/−^ (top) or LDLr^−/−^ (bottom) mice by cardiac puncture. Plasma was separated from the blood cells and pooled plasma samples (n = 4–5 animals) were separated by density gradient ultracentrifugation. Cholesterol (TC, left) and triglyceride (TG, right) were measured in each fraction. ▪, HFC; □, LA; ▵, *c-*9, *t-*11 CLA; ▿ *t-*10, *c-*12 CLA; ⋄, CLA Mix.

The increase in plasma cholesterol and triglycerides with *t-*10, *c-*12 supplementation of the LDLr^−/−^ mice was associated with increased cholesterol in the VLDL and LDL fractions (Figure 1). This was the only significant change in profile among the dietary treatments in either mouse strain.

Fasting blood glucose did not differ among dietary groups in the apoE^−/−^ animals (Table 2). In the LDLr^−/−^ animals blood glucose was increased by *t*-10, *c*-12 CLA supplementation compared to HFC but did not reach statistical significance (P = 0.06).

As in our previous study [23], where *t*-10, *c-*12 CLA supplementation markedly increased plasma insulin levels, the insulin levels in the current study were significantly elevated by *t*-10, *c-*12 CLA (16-fold in apoE^−/−^, p<0.05; and 12-fold in LDLr^−/−^) compared to HFC (Table 2). Although the group mean was markedly elevated in the LDLr^−/−^ animals, the variation was also very large (SEM = 3.2 ng/mL) compared to all other groups. This heterogeneity explained the failure to reach statistical significance. Interestingly NEFA levels were not elevated in any of the supplemented groups for either strain. In fact, the LDLr^−/−^ animals with *c*-9, *t*-11 CLA and CLA Mix supplementation had lower NEFA than HFC (p<0.01) (Table 2).

Adiponectin is an abundant plasma adipocytokine, in non-obese animals and humans, where it is present at approximately 10–20 µg/mL [32]. Lower levels of adiponectin are often associated with obesity and insulin resistant states. *t*-10, *c*-12 CLA supplementation in both the apoE^−/−^ and LDLr^−./−^ mice dramatically reduced plasma adiponectin levels (Table 2). Adiponectin was reduced by approximately 90% with *t*-10, *c*-12 CLA supplementation (p<0.001 and p<0.01 in apoE^−/−^ and LDLr^−/−^, respectively) and by over 50% with the CLA Mix (P = 0.06) supplementation. This isomer-specific reduction has been observed previously [23], [33], [34] and appears to precede any decrease in fat mass [35].

Since *t*-10, *c*-12 CLA supplementation induced significant liver steatosis and hepatomegaly, and marked changes in plasma adiponectin and insulin levels (Table 2), a three week study was undertaken. Poirier *et al* [35] have previously examined the chronology of alterations in adipose tissue, liver weight, hepatic TG accretion, plasma insulin levels and circulating adiponectin in the C57BL/6J mouse. Even though our mouse model (LDLr^−/−^) is on a C57BL/6J background, since the metabolic dysregulation occurs in the absence of a functional LDL receptor, it was imperative to establish the chronology for hepatic and plasma measurements. Changes in body weight did not differ among the diet groups during the first 3 weeks of diet manipulation (data not shown). At 3 weeks, liver weight in the *t*-10, *c*-12 CLA supplementation group showed significant hepatomegaly (Table 3; p<0.001). Conversely, both HFC and *c*-9, *t*-11 CLA supplemented mice had liver weights similar to chow fed animals (3.6±0.1% body weight; n = 3).

**Table 3:** Plasma Measurements for LDLr^−/−^ mice.

		Diet Supplementation			
		HFC	*c*-9, *t*-11CLA	*t*-10,*c*-12CLA	CLA Mix
Initial weight (g)		18.3±0.4	18.2±0.4	17.2±0.4	18.2±0.3
Weight change (g)		2.3±0.4	2.7±0.6	1.9±0.3	2.3±0.5
Liver weight(% body weight)		3.2±0.2	3.5±0.1	9.7±0.8***	4.8±0.5
Cholesterol (mM)	Week 1	12.2±1.2	11.7±1.1	8.8±0.7	9.0±0.4
	Week 3	29.8±0.8**	21.2±1.7	31.0±1.9	21.8±2.5
Triglycerides (mM)	Week 1	2.0±0.4	2.8±0.8	1.4±0.0	1.2±0.1
	Week 3	6.1±1.7*	3.1±0.2	9.6±1.2	4.2±0.9
Adiponectin (µg/mL)	Week 1	19.7±3.7	15.4±2.7	4.3±0.8	9.5±2.6
	Week 3	49.6±10.6	32.7±11.0	3.0±0.2**	20.6±4.2
Insulin (ng/mL)	Week 1	0.7±0.1	1.2±0.2	0.6±0.0	1.2±0.3
	Week 3	1.0±0.1	1.4±0.2	1.6±0.3	1.4±0.2

Mice were fed a HFC diet either supplemented or not with 0.5% fatty acid for 3 wk. Data are mean ± S.E.M. (n = 5–8). ^*^ p<0.05 vs. HFC, ^**^ p<0.01 vs. HFC and ^***^ p<0.001 vs. HFC.

At 0, 1 and 3 weeks of diet intervention blood glucose, plasma adiponectin, insulin, TG and TC were measured. There were no changes in blood glucose among the diet groups (data not shown), but as early as three days after initiation of the test diets, plasma adiponectin was decreased in the *t*-10, *c*-12 CLA diet group (data not shown) and by one week was reduced by approximately 80% compared to HFC (Table 3; p<0.001). Interestingly, after an initial decrease compared to the chow group at 1 week, the HFC, *c*-9, *t*-11 CLA and CLA Mix diet groups all showed an increase in plasma adiponectin at 3 weeks (Table 3). The changes in adiponectin with *c*-9, *t*-11 CLA supplementation were not significantly different from HFC. Plasma insulin was increased by both the *t*-10, *c*-12 CLA and CLA Mix diets by 1 week and continued to increase over 3 weeks of supplementation. These observations indicate that alterations in adiponectin occur very early and are independent of weight loss.

### CLA –associated Changes in Liver Lipids

In our previous study [23], utilizing apoE^−/−^mice, the increased liver weight in the *t*-10, *c*-12 CLA dietary group was accompanied by a nearly three-fold increase in lipid mass, predominantly TG. In the current study, hepatic TG with *t*-10, *c*-12 supplementation was approximately 30% higher in apoE^−/−^ animals and 77% higher in the LDLr^−/−^ compared to HFC (p<0.01). In the apoE^−/−^ mice, but not the LDLr^−/−^ mice, CLA Mix supplementation reduced both hepatic FC and CE compared to the HFC dietary group (Table 4) (p<0.05 and p<0.01, respectively).

**Table 4:** Liver lipid mass for ApoE^−/−^ and LDLr^−/−^ mice.

ApoE^−/−^	Diet Supplementation					
	HFC	LA		*c*-9, *t*-11 CLA	*t*-10, *c*-12 CLA	CLA Mix
TG (mg/g liver)	194.0±15.8	132.2±11.7		148.8±17.9	264.0±14.8**	242.9±12.7
FC (mg/g liver)	4.2±0.2	4.7±0.2		4.0±0.2	3.8±0.2	3.5±0.1*
CE (mg/g liver)	22.6±1.6	21.7±1.8		17.2±1.7	18.5±2.2	9.5±0.8**
PL (mg/g liver)	10.1±0.4	10.9±0.4		9.6±0.4	8.3±0.3**	9.2±0.4
**LDLr^−/−^**	**Diet Supplementation**					
	**HFC**		**LA**	***c*** **-9, ** ***t*** **-11 CLA**	***t*** **-10, ** ***c*** **-12 CLA**	**CLA Mix**
TG (mg/g liver)	40.4±14.5		16.2±3.6	55.5±10.1	176.5±20.8**	67.1±10.3
FC (mg/g liver)	4.9±0.5		5.5±0.4	4.4±0.2	4.9±0.3	4.2±0.3
CE (mg/g liver)	8.0±1.9		5.3±0.8	13.0±2.0	19.5±2.2**	8.8±1.4
PL (mg/g liver)	8.6±0.8		9.4±0.4	10.4±0.3	7.8±0.5	9.8±0.4

Mice were fed a HFC diet either supplemented or not with 0.5% fatty acid for 11 wk. Livers were obtained at sacrifice and lipids extracted in chloroform. TG, FC and CE were quantified by gas-liquid chromatography and PL by phospholipid C enzymatic assay. Data are presented as total mass per gram of liver weight (mean ± S.E.M.; n = 10 to 13). (TG, triglycerides; FC, free cholesterol; CE cholesterol esters; PL phospholipids). * p<0.05 vs. HFC and ** p<0.01 vs. HFC.

### Quantification of Atherosclerosis

**Figure 2:**
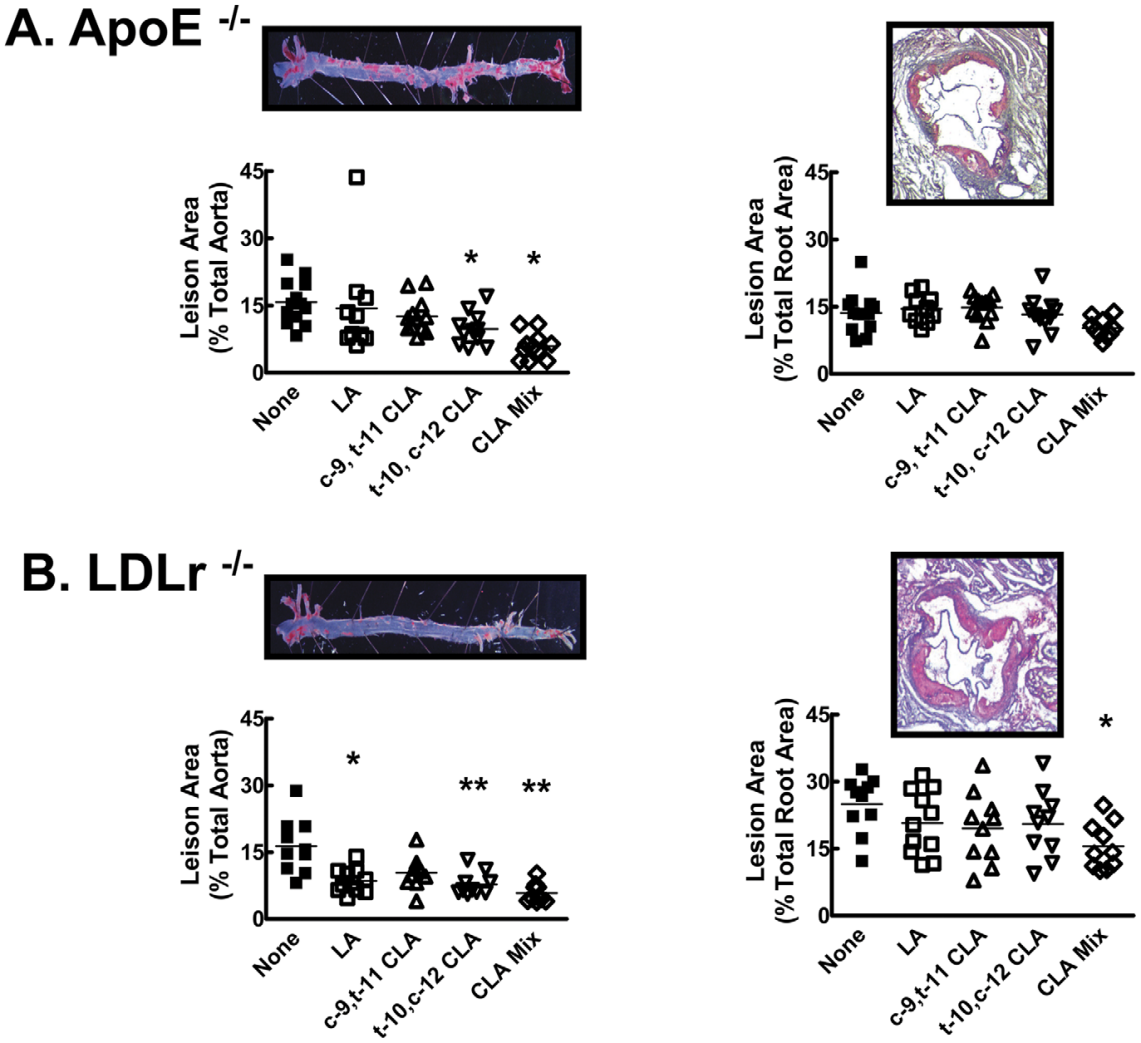
Atherosclerotic lesion measurement in aortic *en face* preparations and aortic root cross sections. The images show representative *en face* (left) and aortic root cross-section preparations (right) from apoE^−/−^ (A) or LDLr^−/−^ (B) mice. Mice were fed HFC diet with or without supplementation for 11 weeks as described in Figure 1. Quantification of percent lesion area in the *en face* preparations was calculated relative to total aorta area. Quantification of cross-sectional area occupied by lesion in the aortic root was determined relative to total cross-sectional area. * vs. HFC, p<0.05; ** vs. HFC, p<0.01.

Atherosclerosis was assessed directly by quantifying lipid staining in *en face* preparations along the length of the aorta from the aortic arch to the iliac bifurcation (Fig. 2, left panels) and in aortic root cross-sections (Fig. 2, right panels). After consuming HFC for 11 weeks, the LDLr^−/−^ animals (Fig. 2B, right panel) had approximately 7% more lesion area in the aortic root than the apoE^−/−^ animals (Fig. 2A, right panel) but only a 2% greater *en face* lesion area (Fig. 2, left panels). Regardless of strain, the animals in the HFC group had the most extensive atherosclerotic lesions, while the diet supplemented with the CLA Mix had the least lesion area, in both the aortic root and in *en face* preparations.

In the LDLr^−/−^ strain, *en face* lesion area was reduced by CLA Mix (5.8±0.7% area, p<0.01), *t*-10, *c*-12 CLA (7.8±0.8% area, p<0.01) and LA (8.6±0.8% area, p<0.05) compared to the HFC group (16.3±1.9% area) (Fig. 2B, left panel). In contrast, there were no significant differences in the extent of aortic root lesions between dietary treatment groups (Fig. 2B, right panel).

The apoE^−/−^ mice showed a similar pattern of lesion development, in that both the CLA Mix (p<0.001) and the *t*-10, *c*-12 CLA (p<0.05) dietary groups had lower lesion area than the HFC group in the *en face* preparations (Fig. 2A, left panel). Again, there were no changes in the aortic root lesions with dietary supplementation (Fig. 2A, right panel).

### Principal Component Analysis of Cardiovascular Risk Factors

**Figure 3:**
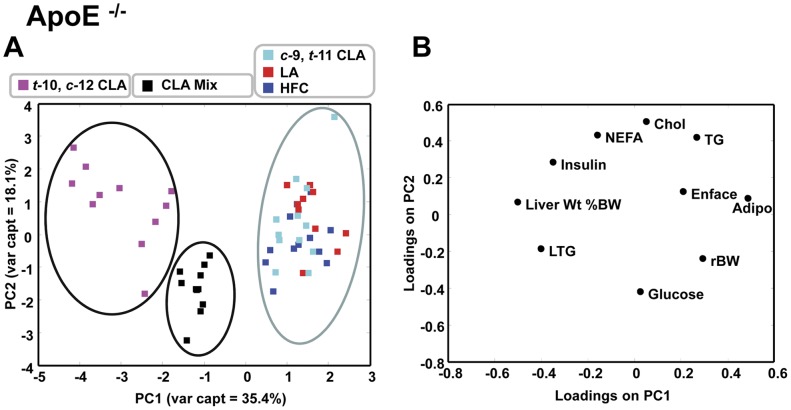
Principal component scores plot of liver and plasma metabolites in apoE^−/−^ mice fed fatty acid supplemented HFC diet (n = 56). (A) The *t-*10, *c-*12 CLA diet exhibits a distinctly different response from the CLA mix and *c-*9, *t-*11 CLA; LA; HFC diets respectively, based on the variables shown in the loadings plot. (B) The loadings plot corresponding to the scores in (A) showing variables that distinguish diets into their respective groups as they appear in the scores plot. See text for details.

**Figure 4:**
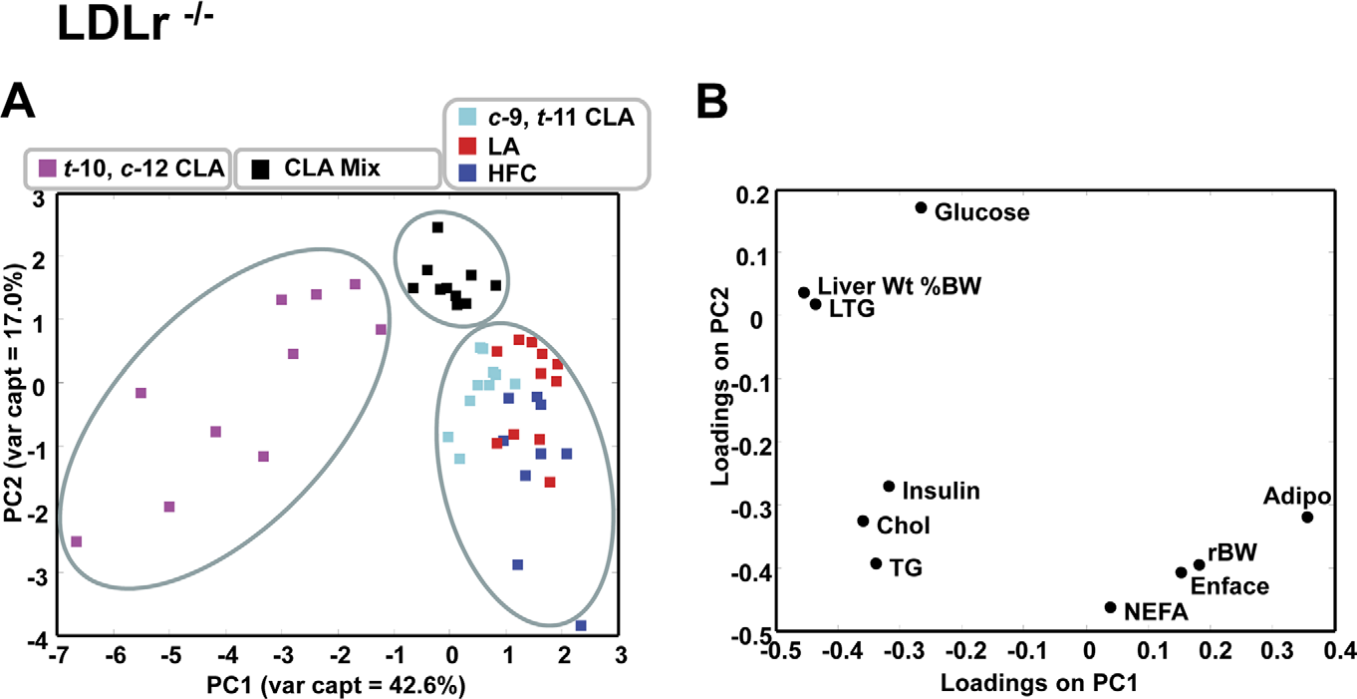
Principal component scores plot of liver and plasma metabolites in LDLr^−/−^ mice fed fatty acid supplemented HFC diet (n = 50). (A) There are three distinct clusters separated along the PC1, corresponding to: *t-*10, *c-*12 CLA; CLA mix; and *c-*9, *t-*11 CLA, LA, HFC diets respectively. (B) The loadings plot corresponding the scores plot shown in (A), depicting the variables that are significant for each cluster as they appear in (A).

**Figure 5:**
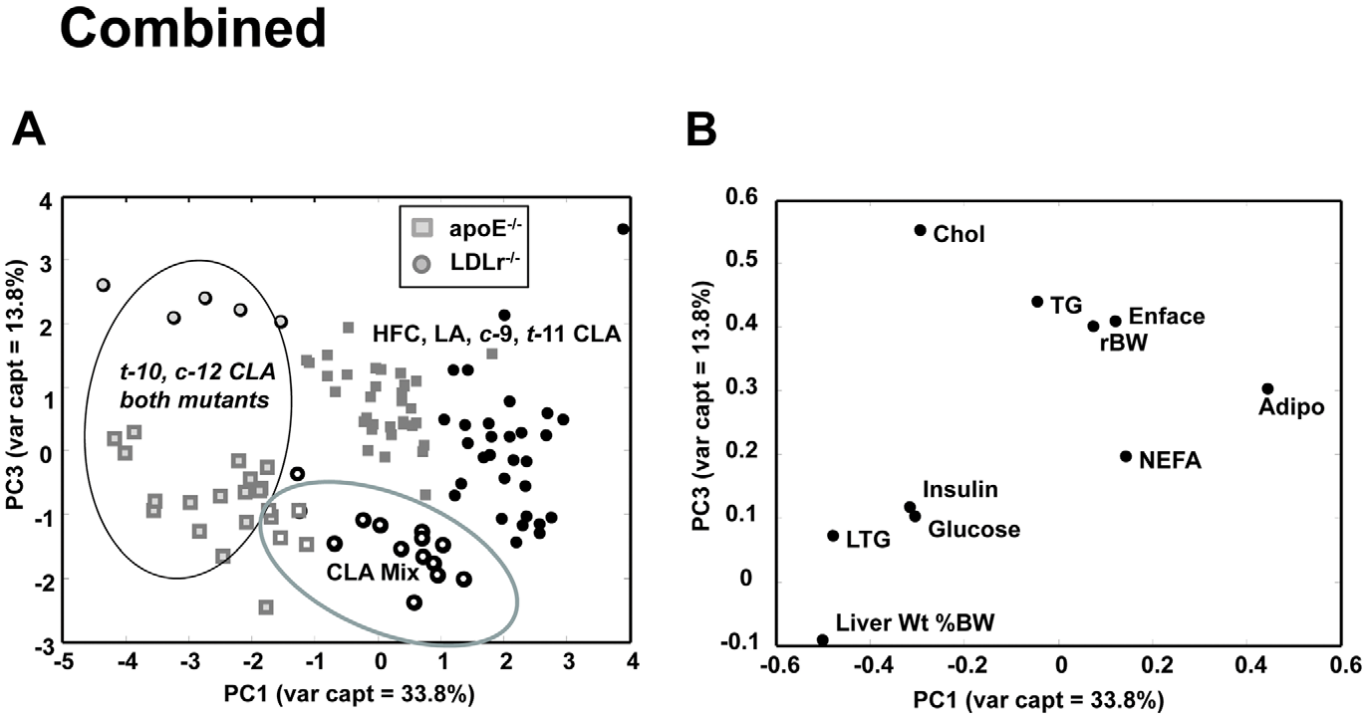
Combined principal component analysis of apoE^−/−^ and LDLr^−/−^ mice fed fatty acid supplemented HFC diet (n = 106). (A) and (B) are the scores and corresponding loadings plots respectively. See text for interpretation.

Risk factor analysis for clinical endpoints can be used to predict the likelihood of complex chronic diseases such as atherosclerosis or metabolic syndrome. In the current study a number of molecular variables were assessed in each mouse strain and relationships among dietary treatment groups were evaluated by PCA. The results from the PCA of cardiovascular and metabolic markers are shown in Figures 3, 4 and 5. The apoE^−/−^ and LDLr^−/−^ mice were first analyzed separately (Figs. 3 and 4, respectively) and then as a combined group (Fig. 5).

For the apoE^−/−^ group the first three principal components (PC1, PC2, PC3) explained 67% of the total variance with PC1 and PC2 responsible for 35% and 18% respectively (Fig. 3A). The dietary treatment groups separated into three distinct groups based on PC1 and PC2. The *t*-10, *c*-12 CLA supplemented animals, which included all animals with substantially elevated plasma insulin, were distinct from all other animals (Fig. 3A, left). The CLA Mix supplemented animals clustered together (Fig. 3A, middle) and loadings analysis indicated that these groupings were delineated by liver TG content, plasma insulin and liver weight (Fig. 3B). The third cluster contained control, LA and *c*-9, *t*-11 CLA supplemented animals (Fig. 3A, right).

For the LDLr^−/−^ group (Figure 4) the first three principal components explained 69% of the total variance and PC1 and PC2 were responsible for 60% of the total variance (43 and 17%, respectively) (Figure 4A). PC1 discriminated between dietary supplementation groups primarily on the basis of plasma and liver TG levels, plasma cholesterol and insulin and liver weight (Fig. 4B). Three distinct groups of animals are discriminated along PC1: all *t*-10, *c*-12 CLA animals clustered on the left (Fig. 4A) while all other dietary groups clustered on the lower and upper right. The Mix diet group also segregated from the other diet groups (Fig. 4A, upper right). Segregation of CLA Mix from *c*-9, *t*-11 CLA, LA and HFC diet supplementation groups seemed to be based on liver weight, liver TG and plasma glucose along the PC2 loading axis (Fig. 4B).

Combined PC analysis also revealed that regardless of mouse strain, the *t*-10, *c*-12 CLA and Mix CLA supplemented animals are distinct from all other diet groups on the basis of liver weight, liver TG and plasma insulin and glucose values (Figure 5). The first three principle components captured 67% of the total variance, with 34% captured by PC1, 19% by PC2 and 14% by PC3. Examination of PC1 versus PC3 axes revealed that the HFC, *c-*9, *t-*11 CLA and LA supplemented mice clustered together in this space, based on plasma TG, NEFA, adiponectin, as well as body weight and lesion size. The animals supplemented with *t-*10, *c-*12 CLA and the mixed isomer preparation were clustered to the lower left of the figure primarily by the influence of liver weight and liver TG content.

### Liver Fatty Acid Analysis

Although it has been previously reported that stearoyl CoA desaturase-1 (SCD1) expression is down-regulated by CLA supplementation [36], [37], we found that each of the individual isomers as well as the CLA Mix increased SCD1 mRNA in the LDLr^−/−^ animals, although the changes were not significantly different from the HFC (data not shown). Nevertheless, fatty acid analysis of the livers (Table S2) showed that the ratio 18∶1/18∶0 was increased 3-fold with *t-*10, *c-*12 CLA supplementation of the HFC (p<0.01), consistent with upregulation of SCD. In contrast, the 16∶1/16∶0 ratio was unchanged.

## Discussion

In the current study we compared two common mouse models of atherosclerosis to assess the influence of two distinct CLA isomers on measurable atherosclerosis and on multiple plasma and tissue markers of atherosclerosis and metabolic dysfunction. An important goal of the current study was to evaluate the ability of potential biomarkers of atherosclerosis and metabolic dysfunction to identify animals at risk of developing adverse metabolic phenotypes based on dietary manipulation. A number of plasma markers of metabolic dysfunction were measured at sacrifice and statistical analysis of the data revealed some significant changes that could contribute to the changes in atherosclerosis. Principal component analysis suggests that several of the changes may cluster around one metabolic factor. Particularly important were the changes that we observed in the liver.

Principal component analysis was performed first within each mouse strain and then with data grouped regardless of strain. This analysis indicated clear differences among diet treatment groups. Regardless of strain, the pooled data indicated that the *t-*10, *c-*12 CLA supplemented group clustered on the basis of liver weight and TG content (Fig. 5), and similar diet group clustering was evident within strain (Figs. 3 and 4). These metabolic parameters may be strong indicators of hepatic insulin resistance. This conclusion is consistent with the increases in plasma insulin that were found by ANOVA (Table 2).

Although we found evidence of reduction in atherosclerosis with *t-*10, *c-*12 CLA isomer and the CLA Mixed isomer supplementation (Fig. 2), there was little support for a role of plasma lipid risk factors in explaining this reduction. As expected for the apoE^−/−^ mouse, which is a severe model of hyperlipidemia, plasma TC was elevated on the HFC diet [38], with typical elevations in VLDL-C in all dietary groups. The influence of various CLA isomers on plasma TG and TC reported in the literature has been inconsistent. In the apoE^−/−^ mouse, published reports indicate no change in TC with a mixed isomer preparation [39]–[41], a decrease with the *c*-9, *t*-11 CLA isomer [42], and an increase with the *t*-10, *c*-12 CLA isomer [23], [42]. In the current study we found no change with the *c*-9, *t*-11 CLA isomer, and a tendency to decrease with the CLA Mix preparation. Dietary cholesterol content in the current study (0.12%, w/w) was lower than in our previous work in the apoE^−/−^ animals [23] (1.25%, w/w), but had little effect on plasma cholesterol levels in the HFC, LA and *c-*9, *t-*11 CLA groups. In contrast, addition of *t-*10, *c-*12 CLA to the HFC diet increased plasma TC, VLDL and LDL in the LDLr^−/−^ mouse. We conclude that CLA has little effect on plasma lipoproteins in the apoE^−/−^ mouse and has a negative impact on plasma lipoproteins in the LDLr^−/−^ mouse. Nevertheless, in both strains, the *t-*10, *c-*12 CLA isomer supplementation reduced atherosclerosis.

Hypoadiponectinemia and hyperinsulinemia are both associated with a pro-atherogenic state, and the previously reported reduction in plasma adiponectin levels by *t*-10, *c*-12 CLA [33], [34], [43] was clearly evident in the current study. Adiponectin was nearly undetectable in the *t*-10, *c*-12 CLA group and substantially reduced in the CLA Mix group. In the principal component analysis, loadings of adiponectin were always associated with the groups that did not contain the *t-*10, *c-*12 CLA isomer, suggesting that this metabolite is a clear discriminator of the groupings. Divergent actions for each CLA isomer have been reported and CLA Mix preparations, depending on the proportion of each isomer, do not have intermediate actions [44]–[46]. In the present study, adiponectin levels in the animals fed the CLA Mix diet were 70% lower than with the *c*-9, *t*-11 CLA isomer and the segregation of Mix from both the *t-*10, *c-*12 CLA cluster and the remaining diets was clear. Thus, it appears that for some metabolic markers the 50∶50 CLA Mix has effects that more closely resemble dominant actions of the *t*-10, *c*-12 CLA isomer.

Adiponectin was decreased and insulin was increased markedly by *t*-10, *c*-12 CLA supplementation in both the apoE^−/−^ and the LDLr^−/−^ mice, so it was surprising that the aortic lesions in the *en face* preparations were significantly reduced compared to HFC in both mouse strains. Mix CLA supplemented animals had a more favourable lipid profile, less severe hypoadiponectinemia and hyperinsulinemia, and also had the largest reduction in aortic lesion development. While the changes in atherosclerosis were evident in *en face* preparations, lesions in the aortic root were unaffected, which may indicate that the root area is the most severely affected and may be therefore resistant to the effects of CLA supplementation. Improvements in atherosclerosis lesions were not observed in our previous study in apoE^−/−^ mice on a diet containing 1.25% cholesterol [23]. This difference may also reflect the severity of the lesions in our earlier work. Reduction of dietary cholesterol did, however, affect lesion development in the apoE^−/−^ mice. In our earlier work, HFHC groups with or without supplementation had approximately 20–25% of the total aorta (*en face* preparation) covered with atherosclerotic lesions [23], whereas in the current study, with 0.12% cholesterol, *en face* lesion staining occupied between 6–15% of the total aorta. Affected aortic root cross-sectional area was also decreased with lower dietary cholesterol, from approximately 40% to 15%. The apoE^−/−^ mouse develops lesions even on a chow diet [47] and dietary cholesterol content enhances lesion development in the early stages. After lesions are established, they are indistinguishable between groups receiving different levels of dietary cholesterol [47]. Therefore, our observations suggest that *t-*10, *c-*12 CLA isomer and CLA Mixed isomer supplementation may be more effective at preventing lesion development than lesion regression.

Few studies have reported on the effect of any CLA isomer supplementation in the LDLr^−/−^ mouse. HFC and HFC with *c*-9, *t*-11 or LA supplementation was associated with lesions covering approximately 10–15% of the total aorta and this was significantly reduced by *t*-10, *c*-12 CLA and CLA Mix supplementation. The LDLr^−/−^ aortic root cross-sections showed more extensive lesions than the apoE^−/−^ mice by almost 10%. Thus, *t-*10, *c-*12 CLA isomer supplementation similarly affected lesion development in two divergent mouse models of atherosclerosis.

There are many discrepancies in the literature regarding the effectiveness of CLA isomers at reducing atherosclerosis and risk factor markers (reviewed in [17]). The *c*-9, *t*-11 CLA isomer was more effective in some studies [40], [42], [48]–[51]. One reason for discrepancies between studies that have used the same animal model may be differences in the base diet used, which can play a significant role in lesion development. It has been previously reported that a semi-purified diet will induce lesion development under less severe dietary manipulations of cholesterol and fat content than a chow-based diet [49], [52]. Using a semi-purified diet supplemented with 1% CLA isomer and 0.15% cholesterol, Arbones-Mainar *et al* [42] showed that the affected lesion area decreased with *c*-9, *t*-11 CLA supplementation while the *t*-10, *c*-12 CLA isomer increased lesion area. Future work will be needed to address the significance of these differences in base diet composition.

The modest improvements in plasma lipid and lipoprotein profiles and atherosclerosis that we observed with *t*-10, *c*-12 CLA supplementation are overshadowed by significant negative changes, particularly in the liver. Our observations support previous reports that this isomer is responsible for decreased weight gain, increased liver weight, diminished adiponectin levels, hyperinsulinemia and the development of hepatic steatosis [3], [33], [35], [43], [44], [53]–[56]. These characteristics are nearly ameliorated by the addition of an equimolar amount of *c*-9, *t*-11 CLA, as observed in the hepatic and plasma metabolite profiles of the CLA Mix supplemented mice. We anticipated that the significant increase in liver weight would be due to accretion of hepatic TG as previously reported [23], [33], [53], [54], [57]. Hepatic TG accumulation was also predicted based on the observation that *t*-10, *c*-12 CLA supplementation resulted in hyperinsulinemia with moderately elevated NEFA levels. Although the increase in hepatic TG was not as profound as with HFHC diet (3-fold increase) [23], hepatic TG was significantly increased compared to HFC, LA and *c*-9, *t*-11 CLA groups. The increases in hepatic TG were accompanied by significant increases in hepatic oleic acid and increases in the hepatic 18∶1 to 18∶0 fatty acid ratio in the LDLr^−/−^ mice, suggesting that the *t*-10, *c*-12 CLA supplementation increases stearoyl-CoA desaturase activity. This change can explain the hepatic TG accumulation, but is unlikely to be responsible for the profound changes in liver weight. Importantly, the 3 week feeding study in these animals provides evidence that profound changes in plasma adiponectin mass and liver weight occur very early after the dietary intervention. Clearly the two CLA isomers have divergent mechanisms of action, as previously documented [42], [44], [58]. Hepatic transcriptome studies conducted in the ApoE3 Leiden mouse [4] suggested that three phases of adaptation may occur in the liver in response to high fat feeding. The early response phase was shown to involve the induction of genes typical of lipid metabolism PPARα, suggesting an increase in β-oxidation to accommodate the increased flux of fatty acids to the liver. Increased expression of PPARγ, another reported target of the CLA isomers [59]–[61], and SCD1 mRNA, indicated an adipogenic transformation of the hepatocytes [4]. PPARγ, may also have anti-diabetic effects, as observed in some models [59]. SREBP is also involved in lipogenesis and its dysregulation has been associated with hepatic steatosis. Changes in these modulators in the transcriptome and proteome of the liver over time may be signals of the change from physiological to pathological accommodation.

It has been demonstrated that the individual CLA isomers are potent ligands and activators of PPARα [62]. The phenotype of the *t*-10, *c*-12 CLA supplemented animal resembles that of a PPARα knockout animal with increased hepatic TG (especially after fasting [63]), increased VLDL production and increased plasma TG [64]. Adiponectin may also increase insulin sensitivity in liver and muscle via PPARα signalling pathways [65], [66]. A lack of PPARα activation, secondary to reduced levels of adiponectin, may provide a potential mechanism for increased insulin resistance in the *t*-10, *c*-12 CLA supplemented mice. This may also explain the differences between the effects of this isomer, the *c*-9, *t*-11 CLA isomer and the mixed isomer preparation. Further work on these potential mechanisms of CLA action is warranted.

Taken together these observations suggest that non-traditional biomarkers may be used to predict changes in atherosclerotic lesions in mouse models. Further refinements of this analysis and extension from animal models to human populations are warranted.

## Supporting Information

Table S1 **Fatty acid composition of experimental diets (wt %, mean ± S.D., n = 3).**
(DOCX)

Table S2 **Fatty acid composition of liver tissue from LDLr^−/−^ mice fed a HFC either supplemented or not with 0.5% fatty acid for 11 weeks.** Livers were obtained at sacrifice and lipids extracted in chloroform. Data are presented as means ± S.E.M., n = 6–10 animals per diet group. ** p<0.01 vs. HFC; *** p<0.001 vs. HFC.
(DOCX)
