# Supplementary material for: t-10, c-12 CLA Dietary Supplementation Inhibits Atherosclerotic Lesion Development Despite Adverse Cardiovascular and Hepatic Metabolic Marker Profiles
Source: PLoS One. 2012 Dec 20;7(12):e52634. doi: 10.1371/journal.pone.0052634 (PMC3527580; doi:10.1371/journal.pone.0052634)
Supplement: Table S1 — Fatty acid composition of experimental diets (wt %, mean ± S.D., n = 3). (DOCX) [file pone.0052634.s001.docx]

| **Supplementary Table 1.** Fatty acid composition of experimental diets (wt %, mean ± S.D., n=3).   \| **Diet Supplementation** \| \| \| \| \| \| \| --- \| --- \| --- \| --- \| --- \| --- \| \| **Fatty acid** \| **HFC** \| **LA** \| ***c*-9, *t*-11 CLA** \| ***t*-10, *c*-12 CLA** \| **CLA Mix** \| \| 16:0 \| 22.5 ± 0.2 \| 21.0 ± 0.8 \| 21.1 ± 0.7 \| 20.6 ± 2.7 \| 20.5 ± 1.1 \| \| 18:0 \| 30.6 ± 1.1 \| 27.9 ± 1.5 \| 25.6 ± 2.9 \| 27.4 ± 3.4 \| 26.1 ± 4.7 \| \| 18:1 (n-9) \| 35.8 ± 7.0 \| 35.3 ± 7.4 \| 37.4 ± 9.8 \| 32.8 ± 6.4 \| 36.7 ± 10.2 \| \| 18:2 (n-6) \| 5.9 ± 0.7 \| 9.5 ± 2.5 \| 6.2 ± 1.2 \| 5.3 ± 0.6 \| 6.0 ± 1.2 \| \| 18:3 (n-3) \| 0.2 ± 0.3 \| 0.4 ± 0.4 \| 0.2 ± 0.3 \| 1.2 ± 1.6 \| 0.5 ± 0.1 \| \| *c*-9, *t*-11 CLA \| nd \| nd \| 4.0 ± 1.5 \| nd \| 1.0 ± 0.2 \| \| *t*-10, *c*-12 CLA \| nd \| nd \| nd \| 2.6 ± 0.9 \| 1.0 ± 0.2 \| \| 20:0  20:1 (n-9) \| 2.2 ± 2.8  1.4 ± 2.4 \| 2.3 ± 3.0  1.9 ± 2.4 \| 2.1 ± 3.7  1.8 ± 3.1 \| 2.0 ± 3.5  2.5 ± 2.4 \| 3.9 ± 3.7  2.7 ± 3.8 \| |
| --- | --- | --- | --- | --- | --- | --- | --- | --- | --- | --- | --- | --- | --- | --- | --- | --- | --- | --- | --- | --- | --- | --- | --- | --- | --- | --- | --- | --- | --- | --- | --- | --- | --- | --- | --- | --- | --- | --- | --- | --- | --- | --- | --- | --- | --- | --- | --- | --- | --- | --- | --- | --- | --- | --- | --- | --- | --- | --- | --- | --- |

nd, not detected
