# Supplementary material for: t-10, c-12 CLA Dietary Supplementation Inhibits Atherosclerotic Lesion Development Despite Adverse Cardiovascular and Hepatic Metabolic Marker Profiles
Source: PLoS One. 2012 Dec 20;7(12):e52634. doi: 10.1371/journal.pone.0052634 (PMC3527580; doi:10.1371/journal.pone.0052634)
Supplement: Table S2 — Fatty acid composition of liver tissue from LDLr−/− mice fed a HFC either supplemented or not with 0.5% fatty acid for 11 weeks. Livers were obtained at sacrifice and lipids extracted in chloroform. Data are presented as means ± S.E.M., n = 6–10 animals per diet group. ** p<0.01 vs. HFC; *** p<0.001 vs. HFC. (DOCX) [file pone.0052634.s002.docx]

**Supplementary Table 2.** Fatty acid composition of liver tissue from LDLr^-/-^ mice fed a HFC either supplemented or not with 0.5 % fatty acid for 11 weeks. Livers were obtained at sacrifice and lipids extracted in chloroform. Data are presented as means ± S.E.M., n=6-10 animals per diet group. ** p<0.01 vs. HFC; *** p<0.001 vs. HFC

| **Fatty Acid** | **Dietary Supplementation** | | | | |
| --- | --- | --- | --- | --- | --- |
|  | **HFC** | **LA** | ***c*-9, *t*-11 CLA** | ***t-*10,*c*-12 CLA** | **CLA Mix** |
| C14:0 | 0.37 ± 0.03 | 0.31 ± 0.02 | 0.36 ± 0.02 | 0.37 ± 0.01 | 0.35 ± 0.01 |
| C16:0 | 20.48 ± 0.89 | 20.82 ± 0.66 | 20.98 ± 0.49 | 23.05 ± 0.46 | 21.51 ± 0.36 |
| C16:1 | 2.23 ± 0.43 | 1.45 ± 0.21 | 2.71 ± 0.18 | 3.18 ± 0.18 | 2.51 ± 0.14 |
| C18:0 | 7.54 ± 0.96 | 10.41 ± 0.58 | 5.95 ± 0.56 | 2.75 ± 0.30 | 5.13 ± 0.38 |
| C18:1 | 32.51 ± 3.03 | 29.69 ± 2.36 | 41.59 ± 2.31 | 51.56 ± 0.82*** | 42.97 ± 2.01 |
| C18:2 | 6.71 ± 0.43 | 8.79 ± 0.31 | 5.74 ± 0.18 | 2.67 ± 0.18 | 5.66 ± 0.20 |
| C20:0 | 0.62 ± 0.04 | 0.66 ± 0.05 | 0.61 ± 0.04 | 0.27 ± 0.02*** | 0.55 ± 0.03 |
| *c*-9*, t*-11 CLA | ND | ND | 0.68 ± 0.04 | ND | 0.32 ± 0.02 |
| *t*-10, *c*-12 CLA | ND | ND | ND | 0.13 ± 0.01 | ND |
| 16:1 /16:0 | 0.11 ± 0.02 | 0.07 ± 0.01† | 0.13 ± 0.01 | 0.14 ± 0.01 | 0.12 ± 0.01 |
| 18:1/18:0 | 5.99 ± 1.57 | 3.03 ± 0.39 | 7.61 ± 0.84 | 20.45 ± 1.86** | 8.85 ± 0.77 |
